# Factors influencing household and women’s dietary diversity in migrant households in central Nepal

**DOI:** 10.1371/journal.pone.0298022

**Published:** 2024-04-05

**Authors:** Hom Nath Gartaula, Kishor Atreya, Kanchan Kattel, Dil Bahadur Rahut

**Affiliations:** 1 Sustainable Impact Department, International Rice Research Institute, Los Banõs, Philippines; 2 School of Forestry and Natural Resource Management, Institute of Forestry, Tribhuvan University, Kathmandu, Nepal; 3 Department of Watershed Management and Environmental Science, Institute of Forestry, Tribhuvan University, Pokhara Campus, Nepal; 4 Department of Nutrition and Public Health, University of Agder, Kristiansand, Norway; 5 Asian Development Bank Institute (ADBI), Tokyo, Japan; University of Greenwich, UNITED KINGDOM

## Abstract

Food security and dietary diversity, defined as providing either physical (availability) or economic (accessibility) access to food, are linked with access to and control over productive resources and is a highly-gendered phenomenon. In Nepal, labor out-migration has increased household income and may have increased people’s ability to access diverse food either by increasing investment in agriculture or purchasing various food items from the market. However, the relationship between household dietary improvement and labor out-migration is complex. Drawing on a survey of 1,053 migrant households in three agroecological regions of Nepal, this paper disentangles this complex phenomenon by showing how household dietary diversity and women’s dietary diversity are influenced by biophysical, social, economic, and cultural factors. The influence of the amount of remittances, land abandonment, and women confined to the house by household chores are some factors that policy makers should consider seriously in designing gender-sensitive nutrition policies. The expansion of women’s agency contributes to enhancing dietary diversity and specifically women’s dietary diversity at the household level; however, how these factors determine an individual’s dietary diversity depends on intrahousehold dynamics and relations.

## 1. Introduction

Food insecurity and limited access to healthy diets are significant and severe problems, especially in low-income countries [1], including Nepal. Despite an increasing trend in the per capita household food consumption, the Nepal Demographic and Health Survey 2022 [2] shows that 13% of Nepalese experience moderate to severe food insecurity, with mountain population experiencing more (21%) food insecurity than terai population (12%). Studies report increasing inclusion of protein (particularly animal protein) and decreasing reliance on cereals and tuber-based diets to increase dietary diversity [3]. An increase in access to diverse food can be attributed to increased purchasing power through remittances sent by migrant workers, which contribute 24% of the national gross domestic product (GDP) [4]. The literature shows a significant increase in calorie consumption and a reduction in headcount food poverty among migrant households [5]. However, the relationship between household dietary improvement and labor out-migration is complex [6]. This is because remittances can alleviate anxiety over having access to enough food. However, it can also come at a cost of unsafe work at the destination, and additional work burden on the women left behind, reflecting gendered processes [7]. Using survey data collected from 1,053 maize-growing households, in this paper we analyze the household dietary diversity and women’s dietary diversity among migrant households in the three agroecological regions of Nepal.

The household dietary diversity score (HDDS), a proxy indicator for household access to various food items, measures household-level dietary diversity. Similarly, the women’s dietary diversity score (WDDS) is a proxy measure for women’s access to diverse diets. The WDDS stands for the adequacy of household diet in terms of macro- and micronutrients most needed by women, especially during reproductive age [8]. The HDDS is generally used to assess household food security, while the WDDS is used to validate individual women’s nutritional adequacy within households, adding combined analysis. A positive association of the HDDS with higher socioeconomic status and food security of households is evident [9, 10]. The relationship between the HDDS and the WDDS has been well established with farm production diversity [11–13], market purchase of food [14, 15], assets, wealth, and education [16, 17]. The two diversity scores (HDDS and WDDS) are also related; for example, a study in Niger [18] found that a unit increase in the HDDS contributes to a 0.396 unit increase in the WDDS. However, the relationship could differ in diverse social and cultural contexts, mainly where male labor out-migration is significant, which can influence food access and consumption of a nutritious diet between men and women within a household.

**Fig 1:**
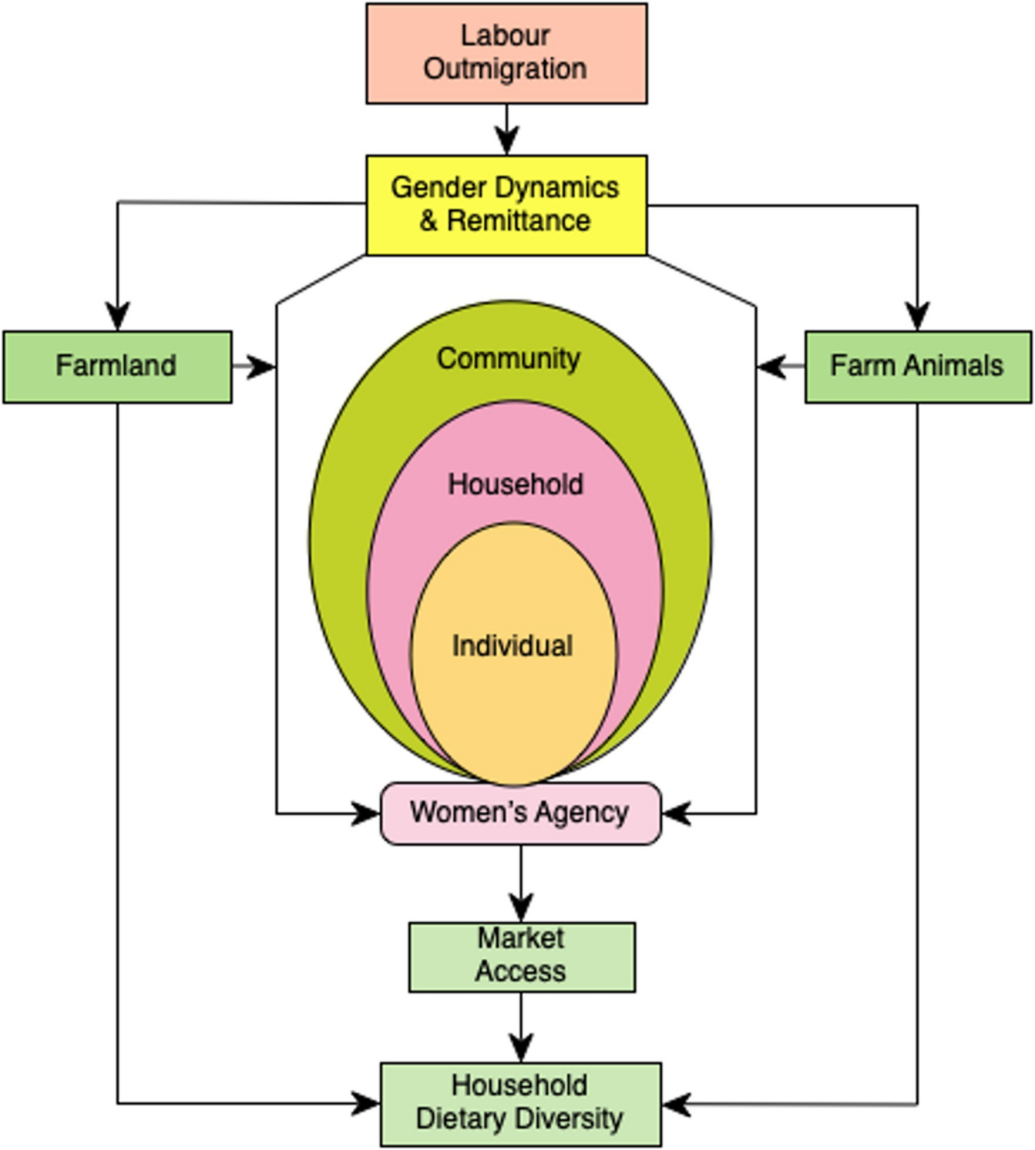
Conceptual framework: Migration–gender–dietary diversity nexus. Source: Authors’ creation.

This paper is framed around the dynamic migration–gender–dietary diversity nexus, which intersects between remittances, agriculture (farmland and farm animals), market access, and women’s agency at the individual, household, and community levels, impacting the dietary diversity of migrant households in general, but of the women left behind in particular (Fig 1). Out-migration changes the intrahousehold gender dynamics, changing women’s agency within the household and community services [19, 20]. Remittances can be used for maintaining or enhancing dietary diversity by changing the agricultural production system, market interaction, and raising small farm animals. However, the mechanism is not as straightforward as it seems [5]. Out-migration possibly improves households’ economic access to market foods [6, 19], but the diversity and quality of food consumed from the market can be poor [21]. Nevertheless, with increased access to remittances and food options in the market, the composition of household food baskets is changing over time [20].

Out-migration may also increase women’s agency, primarily through their increased involvement in household and community activities and the degree of autonomy women assume in household decision making [22, 26–28]. Women with a higher agency are more likely to ensure dietary diversity in their households [23, 24] but not in every context [25]. Thus, not only do remittances influence household dietary diversity [17], but male out-migration through enhanced women’s agency increases women’s standing in society and autonomy to make decisions within households [20]. An enhanced women’s agency is often associated with greater efforts to ensure household dietary diversity for the family [23, 26, 27]. At the same time, gender dynamics within households also play a crucial role in the WDDS, which might differ from the HDDS because of the prevalence of gender-based micronutrient intake disparities in society [28].

In this paper, we assume that remittances enhance women’s agency within and beyond the household, and thus migrant households enhance their dietary diversity. The migration–gender–dietary diversity nexus is, however, rarely studied in the context of the remittance economy [6, 19, 29]. Thus, this study will contribute to the literature (i) by estimating household dietary diversity and women’s dietary diversity in migrant households, and (ii) by identifying factors affecting household dietary diversity and women’s dietary diversity in migrant households. This study offers an interesting illustration of how household dietary intake and nutrition security are affected by the rapidly growing trend of labor out-migration, agricultural activities, and gender dynamics within households in the origin areas.

## 2. Methods

### 2.1 Study area

The study was carried out in three rural municipalities in Nepal: Ichchhakamana of Chitwan district, Gandaki of Gorkha district, and Panchpokhari Thangpal of Sindhupalchok district, representing the terai, hills, and mountain agroecological regions, respectively. These districts can be reached by a 1-day drive from Kathmandu, and the municipalities were chosen because of the greater numbers of the migrant population. Although labor migration is common in these municipalities, there are other differences, especially in socioeconomic and biophysical characteristics (Table 1).

**Table 1:** Geographic and demographic characteristics of the study areas.

Characteristics	Study areas		
Municipality	Gandaki	Panchpokhari	Ichchhakamana
District	Gorkha	Sindhupalchok	Chitwan
Ecological region	Hills	Mountains	Terai (inner terai)
Area (km^2^)	124	187	167
Approximate elevation (meters above sea level)	270 to 1,707	900 to 4,100	500 to 1,800
Population (2019)	32,145 (5,763 households)	34,697 (7,803 households)	26,350 (5,227 households, 2020)
Household economy	Agriculture–commercial vegetables	Agriculture and tourism	Agriculture–commercial vegetables
Main crops	Rice, maize, potato, pulses, and vegetables	Potato in winter, maize, and millet in summer	Rice, maize, potato, pulses, and vegetables
Migration %	36	31	21
Religion	Mainly Hindu, few Christians, and Buddhist	Buddhist (67%), Hindu (31%), and few Christian	Mainly Hindu, few Christians, and Buddhist
Ethnic group composition	Brahmin and Chhetri 25%, Magar 18.3%, Dalits 14.3%, Gurung 14%, Newar 13.3%, Chepang 12.3% and others	Tamang 60%, Chhetri 12.5%, Dalits 6.7%, Newar 5.8%, and remaining others (Holmo, Gurung, Sherpa, Brahmin, Magar)	Chepang 33.3%, Gurung 26.7%, Magar 12%, Brahmin 4.8%, Chhetri 4.5%, Tamang 4%, Newar 3.1% and remaining others (Dalits and Gharti)

Source: Compiled by Authors.

### 2.2 Sampling strategy and data collection method

The study focuses on out-migration and the change in gender roles, and the consequent dietary diversity of women and at the household level. We thus interviewed households with at least one member working abroad during the survey/fieldwork. We first contacted the municipal authorities for the list of migrant households, which was unavailable; thus, we estimated the number of migrant households through the population data available for the districts. The Government of Nepal reported that 21% of the population in Chitwan, 36% in Gorkha, and 31% in Sindhupalchok districts were migrants. We used an average value of 30% for estimating the migrant population in each ward, which was divided by the average family size of five members to estimate the migrant households. Finally, we sampled 20% of the migrant households from 23 wards of the three municipalities for interview (Table 2).

**Table 2:** Ward level household numbers and population, and the sampled households in three municipalities.

Municipality	Ward	Household Number*	Total Population*	Migrant Population**	Migrant Household**	Sample Household***
Icchyakamana (2017)	1	505	3,074	922	184	37
	2	622	3,544	1,063	213	42
	3	652	3,187	956	191	38
	4	720	3,553	1,066	213	43
	5	835	3,982	1,195	239	48
	6	652	3,694	1,108	222	44
	7	794	4,033	1,210	242	48
Gandaki (2018)	1	637	3,581	1,074	215	43
	2	862	4,779	1,434	287	57
	3	591	3,351	1,005	201	40
	4	625	3,397	1,019	204	41
	5	819	4,730	1,419	284	57
	6	794	4,738	1,421	284	57
	7	637	3,305	992	198	40
	8	798	4,264	1,279	256	51
Panchpokhari Thankpal (2018)	1	957	3,975	1,193	239	48
	2	840	4,032	1,210	242	48
	3	954	4,813	1,444	289	58
	4	972	4,708	1,412	282	56
	5	690	3,271	981	196	39
	6	1,207	5,050	1,515	303	60
	7	1,414	5,422	1,627	325	65
	8	769	3,426	1,028	206	41
TOTAL	23	18,346	91,909	27,573	5,515	1,100

Notes:

* Source included are the unpublished reports from the respective municipalities. Respective year in parentheses indicate the year data were available to retrieve.

** Neither the ward levels nor the municipality levels data on migrant population were available; however, such data were available at the district level.

*** We proportionately allocated 20% of the migrant households in each ward for sampling. For household selection for interviews, we applied the snowball sampling technique.

We conducted the household survey between September and November 2020 using a snowball sampling technique [30]. We used this technique to select the sample households for two reasons: first, the list of migrant households (the sampling frame) was unavailable, and second, reaching out to randomly selected households was impractical due to mobility issues during the COVID-19 pandemic. For snowball sampling, first the total sample was proportionately allocated into 23 wards of the three municipalities, and the enumerators asked the respondents to refer to another (nearby) migrant household within the ward and followed up accordingly. A total of 1,053 households were interviewed. Using checklists, we also conducted nine focus group discussions and 13 key informant interviews. Before administering the survey, the questionnaire was translated into the local language and piloted in the non-sampled households in the nearby villages. Well-trained enumerators conducted the interviews using the Android-based Kobo Toolbox for recording the data. Persons aged 18 years and over, available at home during the survey, who are mainly involved in making decisions on households’ daily activities, were interviewed. Before interviews, we informed each respondent of the study objective, anonymity, and confidentiality, and recorded their consent before proceeding. We followed procedure for data collection ethics administered by the International Maize and Wheat Improvement Center’s Institutional Research Ethics Committee (approval number IREC.2020.031).

### 2.3 Estimation of dietary diversity

This paper used the HDDS and the WDDS as proxies for (i) household access to food varieties, and (ii) micronutrient adequacy in their diet, respectively. The scores were calculated using qualitative measures of the dietary intake for a specific period (24 hours recall) with a slightly different categorization of food groups. Even though there is no universal consensus, 12 food groups were used for the HDDS calculations [31] and nine food groups were used for the WDDS [32] calculations. The food groups included in the WDDS calculations reflect the probability of micronutrient adequacy in the household’s diet. We documented the food items eaten by any household member following the Food and Agriculture Organization’s (FAO) Guidelines [8]. The guidelines provide 16 food categories (See Table 3 for detailed classification of food groups). We inquired whether any household member had eaten a particular food item over the previous 24 hours (the previous day—morning to night). For this, we prepared a colorful two-sided single-page checklist of the 16 categories of food items and provided it to each enumerator to show respondents during interviews (S1 Table). The study did not set a minimum quantity of food to be consumed, and even if a small amount of food was consumed by any household member in the 24 hours recall period, we counted it as “Yes” (coded 1), otherwise “No” (coded 0). The scores reflect economic access to a variety of foods [8] and thus show the access dimension of household-level food security. Using the survey data, we calculated the HDDS (sum of the 12 category food groups) and the WDDS (sum of the nine category food groups) (Table 3). We further divided the HDDS into three groups based on the number of food groups consumed following Mekuria et al. [33]:

- low (when households consumed less than or equal to three food groups),
- medium (when households consumed four to six food groups), or
- high (when households consumed more than or equal to seven food groups).

**Table 3:** Categorization of 16 food groups into HDDS and WDDS.

Question number	Food group (16 categories)	Question number	HDDS (12 categories)	Question number	WDDS (9 categories)
1	Cereals	1	Cereals	1, 2	Starchy staples
2	White roots and tubers	2	White roots and tubers		
3	Vitamin A rich vegetables and tubers	3, 4, 5	Vegetables	4	Dark green leafy vegetables
4	Dark green leafy vegetables			3, 6	Other vitamin A rich fruit and vegetables
5	Other vegetables			5, 7	Other fruit and vegetables
6	Vitamin A rich fruit	6, 7	Fruits		
7	Other fruit				
8	Organ meat	8, 9	Meat	8	Organ meat
9	Flesh meat			9, 11	Meat and fish
10	Eggs	10	Eggs	10	Eggs
11	Fish and seafood	11	Fish and seafood		
12	Legumes, nuts, and seeds	12	Legumes, nuts, and seeds	12	Legumes, nuts, and seeds
13	Milk and milk products	13	Milk and milk products	13	Milk and milk products
14	Oils and fats	14	Oils and fats	Not accounted	
15	Sweets	15	Sweets		
16	Spices, condiments, beverages	16	Spices, condiments, beverages		

HDDS = household dietary diversity score, WDDS = women’s dietary diversity score.

Source: FAO Guidelines for Measuring Household and Individual Dietary Diversity (2010).

### 2.4 Data analysis

Besides frequency tabulation and descriptive statistics, we used ordinary least squares (OLS) regressions to identify the influencing factors for the HDDS and the WDDS. The dependent variables are the HDDS (sum of the 12 food groups), ranging from 0 to 12, and WDDS (sum of the nine food groups), ranging from 0 to 9. Most independent variables (Table 4) are self-explanatory; a few more complicated ones (household assets, livestock unit 1, livestock unit 2, women’s involvement in household chores, and women’s involvement in community activities) are explained here. The household asset is the sum of the presence or absence of 15 household physical assets: television, refrigerator, smart mobile phone, traditional non-touch phone, automobile, motorcycle or scooter, bicycle, computer (desktop and/or laptop), power generator, air conditioner, table and/or ceiling fan, power tiller (small tractor), threshing machine, solar hot water system for bathing, and access to an electricity grid line—each coded 1 if the household owned at least one; and coded 0 when absent. Livestock units 1 and 2 are the number of livestock a household owns, converted to a common unit using the FAO conversion factor (cf) for South Asia. Livestock unit 1 is a proxy for the availability of milk and dairy products in the household diet. Livestock unit 1 thus accounted for dairy farm animals—cow (cf 0.5) and buffalo (cf 0.5). Likewise, livestock unit 2 is a proxy for the availability of meat and eggs in the household diet. Thus, livestock unit 2 accounted for pig (cf 0.2), goat (cf 0.1), sheep (cf 0.1), poultry (cf 0.01), and rabbit (cf 0.01).

**Table 4:** Independent variables and their expected influence on HDDS and WDDS.

SN	Variable name	Explanation	Hypothesis	
			HDDS	WDDS
1	Age of the respondent (years).	Younger respondents tend to report higher dietary diversity and include food groups consumed outside the home, thereby reporting higher HDDS and WDDS	-	-
2	Gender of the respondent	Gender of the respondent (male 1, 0 female). Females report higher HDDS and WDDS as it is mostly women involved in cooking and arranging food items in the household.	-	-
3	Respondent is the household head	(yes 1, 0 otherwise). Household head is generally an older male member, and may not involve in food preparation, thus might miss out on some foods included in 24-hour household meals, hence may report lower HDDS and WDDS.	-	-
4	Household size	Number of members, including migrants in the household. Dietary diversity is higher in small and nuclear households [36].	-	-
5	Household Assets	Sum of 15 assets (score value). The dietary diversity increases with the economic status of the household, asset ownership being one indicator [68].	+	+
6	Location: Hill	Dummy for location 1 (Gandaki 1, 0 otherwise). Households in Gandaki are more involved in vegetable farming and are relatively nearby markets, so they are more likely to consume diverse food items.	+	+
7	Location: Mountain	Dummy for location 2 (Pachpokhari 1, 0 otherwise). Mountain regions are fragile, less fertile, and remotely located, thus less diversification of food production and limiting own diversified production and food purchase from markets–ultimately reducing the HDDS and WDDS.	-	-
8	Irrigated land *(Khet)* in hectares (ha)	Cultivated lowland *(Khet)* (irrigated land) (ha). Size of farmland such as lowland *(Khet)* is positively associated with production diversity and hence HDDS and WDDS [69]. Farming different varieties of cereals, vegetables, roots, and tubers in lowland *(Khet)* is possible.	+	+
9	Upland *(Bari)* in hectares	Cultivated *Bari* or unirrigated upland (ha). Having a large proportion of upland *(Bari)* is associated with a lesser variety of crops as the migrant household uses it suboptimally because of labor shortages, negatively affecting the diversity of food produced and consumed.	-	-
10	Upland *(bari)* abandoned	At least one parcel of upland or *Bari* is abandoned permanently. Abandonment of upland is negatively associated with HDDS and WDDS as it may decrease the amount and variety of easily accessible homegrown food.	-	-
11	Household food self-sufficiency	Household food self-sufficiency (own production sufficient for 12 months or more 1, 0 otherwise). HDDS increases with own farm level production diversity [12, 13], so both HDDS and WDDS can be expected to be positively associated with food self-sufficiency.	+	+
12	Frequency of food purchase	How often a household procures food from the market? (1 rarely, 2 occasionally, 3 always). Food purchase provides an opportunity to include food groups other than the ones self-produced to include in the diet and enhance HDDS and WDDS.	+	+
13	Livestock unit 1	Calculated livestock index for milk and dairy products. The index accounts for cows and buffaloes present in the household. Livestock ownership improves dietary diversity through direct consumption of animal products (milk and other dairy products) and increased consumption expenditures [53].	+	+
14	Livestock unit 2	Calculated livestock index for meats. This index accounts for goats, sheep, poultry, rabbits, and pigs in the households. Livestock ownership improves dietary diversity through direct consumption of animal products (egg and meat products) and increased consumption expenditures [53].	+	+
15	Remittance income	Log of remittance income (NPR ‘000). Remittances increase the opportunity to purchase diverse food for consumption, thereby enhancing HDDS and WDDS [19].	+	+
16	Women’s involvement in household chores	Index value for women’s involvement in household chores. Higher values show more involvement. Women’s involvement in household work signifies more effort put into preparing a diverse diet [27]; hence we expect higher HDDS and WDDS.	+	+
17	Women’s involvement in community activities	Index value for women’s involvement in community work. Higher value shows their higher community engagement and mobility outside of the domestic sphere. Women’s involvement in community work signifies their agency and empowerment. Studies have found a positive association between women empowerment and dietary diversity [24, 59].	+	+

HDDS = household dietary diversity score, WDDS = women’s dietary diversity score.

Similarly, women’s involvement in household chores and women’s involvement in community activities are the average value of the 5-point Likert scale responses for women’s involvement in household and community activities, respectively. We estimated women’s involvement in household chores by averaging responses on the six household activities: cleaning, cooking, childcare, elderly care, child education, and operational decisions. For example, we asked each respondent, “who cooks food?” and noted their responses (“Always men = 1,” “Usually men = 2,” “Both men and women = 3,” “Usually women = 4,” “Always women = 5”). Higher values show more involvement of women in daily household work. The higher participation of women in household work, including cooking food, signifies more effort to prepare a diverse meal. Likewise, we estimated women’s involvement in community activities by averaging responses on the four key statements—more women involved in community groups, more women take decisions in the community groups, increased women’s participation in community work, and increased recognition of women as a “farmer.” For example, we asked, “because of labor out-migration, are women more involved in community groups?” and coded their responses on the 5-point Likert scale (“Strongly disagree = 1,” “Disagree = 2,” “Neutral = 3,” “Agree = 4,” “Strongly agree = 5”). A higher value shows more agreement to increased women’s mobility and community engagement, indicating women’s higher agency.

## 3. Results and discussion

In this section, we first present descriptive statistics of the sample households and the respondents’ individual and household characteristics such as landholding, food sufficiency, household income status, food items consumed, and the HDDS and the WDDS. The second part of this section discusses how these dietary diversity scores are influenced by different independent variables organized in different ways. We looked at the influence of the respondent’s individual (particularly age and gender) and household characteristics (farm size, household assets, location, land and food dynamics, large and small livestock, and finally remittances and women’s agency). This helped us to unpack the nuances of this complex migration–gender–dietary diversity nexus and understand its policy implications in a more systematic manner.

### 3.1 Profile of the sample households

Table 5 shows that, on average, two-thirds of the respondents were women with bare minimum education (50% never went to school, and many others were just literate). Over two-thirds (68%) belonged to the hilly Janajati ethnic groups like Tamang, Gurung, Lama, Shyangbo, Magar, and Dong. The rest (32%) were from other caste groups such as Brahmin, Chhetri, Thakuri, Newar, Dalit, and others. Most households (62%) lived in nuclear families (a couple and their children). The average age of respondents was 45 years, and the household size was 6.5 (including migrants), about an equal male-to-female ratio (Table 6).

**Table 5:** Frequency tabulation of the sample population characteristics.

		Gandaki (n = 394)		Ichchhakamana (n = 228)		Panchpokhari (n = 431)		Total (n = 1,053)	
		No. of HHs	Percent of HHs	No. of HHs	Percent of HHs	No. of HHs	Percent of HHs	No. of HHs	Percent of HHs
Gender	Female	299	75.9	129	56.6	280	65.0	708	67.2
	Male	95	24.1	99	43.4	151	35.0	345	32.8
Education	Never been to school	186	47.2	102	44.7	235	54.5	523	49.7
	Grades 1–8	149	37.8	95	41.7	132	30.6	376	35.7
	Grades 9–12	38	9.6	19	8.3	35	8.1	92	8.7
	Grade >12	21	5.3	12	5.3	29	6.7	62	5.9
Caste/Ethnicity	Brahmin Chhetri Thakuri	73	18.5	35	15.4	45	10.4	153	14.5
	Newar	78	19.8	1	0.4	16	3.7	95	9.0
	Janajati	180	45.7	181	79.4	357	82.8	718	68.2
	Dalit	63	16.0	11	4.8	11	2.6	85	8.1
	Others	0	0.0	0	0.0	2	0.5	2	0.2
Family type	Joint family	161	40.9	107	46.9	132	30.6	400	38.0
	Nuclear family	233	59.1	121	53.1	299	69.4	653	62.0

HH = household.

**Table 6:** Descriptive statistics of respondents’ age and household size.

Variable	Gandaki		Ichchhakamana		Panchpokhari		Total	
	Mean	SD	Mean	SD	Mean	SD	Mean	SD
Age of the respondent	46.7	15.4	45.5	17.9	42.2	14.9	44.6	15.9
Family size	6.7	3.0	7.1	2.9	6.0	2.8	6.5	2.9
Male members	3.4	1.7	3.8	1.7	3.0	1.6	3.3	1.7
Female members	3.3	1.8	3.3	1.6	3.1	1.7	3.2	1.7

SD = Standard Deviation.

### 3.2 Landholding and food sufficiency

Almost all households had unirrigated upland (called *bari* in the local language). Sample households from Ichchhakamana municipality had the largest size of cultivated upland (0.40 hectares [ha]), followed by Gandaki (0.32 ha) and Panchpokhari (0.27 ha) municipalities. However, the distribution of cultivated lowland *(khet)* was not the same between municipalities. Panchpokhari had the highest average cultivated lowland (0.50 ha), and Ichchhakamana households had the lowest average cultivated lowland (0.24 ha). The households in Panchpokhari had the highest average cultivated landholding size (0.61 ha) compared to the other two municipalities (Table 7).

**Table 7:** Households’ landholdings.

Land holdings	Gandaki			Ichchhakamana			Panchpokhari		
	No. of HHs	Area (ha)	SD	No. of HHs	Area (ha)	SD	No. of HHs	Area (ha)	SD
Cultivated *bari* (upland)	388 (98%)	0.32	0.23	216 (95%)	0.40	1.10	427 (99%)	0.27	0.32
Cultivated *khet* (irrigated lowland)	140 (36%)	0.42	0.41	59 (26%)	0.24	0.17	289 (67%)	0.50	0.53
Total	389	0.47	0.43	228	0.44	1.07	428	0.61	0.71

ha = hectare, HH = household, SD = Standard Deviation.

None of the surveyed households in all three municipalities had sufficient food supply from their own production (Table 8). Specifically, about 66% of households in Panchpokhari, 83% in Ichchhakamana, and 91% in Gandaki municipalities did not have sufficient year-round food supply from their own agricultural production. Overall, 20% households had food self-sufficiency, and the rest tended to buy food from the market. About 10% of households reported that they “always” buy food from the market, while 86% purchase “occasionally,” and the remaining 4% “rarely” (Table 8).

**Table 8:** Households’ food sufficiency and market dependency for food purchase.

Variables	Gandaki		Ichchhakamana		Panchpokhari		Total	
	No. of HHs	%	No. of HHs	%	No. of HHs	%	No. of HHs	%
Household food sufficiency								
Insufficient	357	90.6	188	82.5	285	66.1	830	78.8
Sufficient	37	9.4	40	17.5	146	33.9	223	21.2
Total	394	100	228	100	431	100	1053	100
Household dependency on market for food purchase								
Rarely	17	4.3	12	5.3	14	3.2	43	4.1
Occasionally	316	80.2	214	93.9	380	88.2	910	86.4
Always	61	15.5	2	0.9	37	8.6	100	9.5
Total	394	100	228	100	431	100	1053	100

HH = household.

### 3.3 Household income sources

Table 9 shows that almost all the respondents (99%) relied on agriculture as a source of household income, while 89% received earnings from raising farm animals such as goats and poultry, and a similar number of households (89%) stated remittances as one of the major sources of income. We observed that a limited number of households depend on other sources such as petty trade, jobs, business, and private sector jobs for income. Comparatively, 64% of households in Ichchhakamana stated their dependency on wages and labor for earnings compared to Gandaki (32%) and Pachpokhari (43%). In contrast, a higher number of households in Pachpokhari reported their dependency on private sector services and other small businesses (Table 9).

**Table 9:** Percentage of households by income source.

Income source	Gandaki		Ichchhakamana		Panchpokhari		Total	
	Household	%	Household	%	Household	%	Household	%
Agriculture	392	99.5	228	100.0	422	97.9	1042	99.0
Farm animals	356	90.4	214	93.9	365	84.7	935	88.8
Remittances	343	87.1	198	86.8	392	91.0	933	88.6
Wages and labor	127	32.2	145	63.6	183	42.5	455	43.2
Private services	14	3.6	8	3.5	73	16.9	95	9.0
Business	28	7.1	8	3.5	57	13.2	93	8.8
Government services	40	10.2	10	4.4	12	2.8	62	5.9
Petty trade/small shop	19	4.8	24	10.5	11	2.6	54	5.1
Others (unspecified)	1	0.3	0	0.0	1	0.2	2	0.2

### 3.4 Food items in the household diet

**Fig 2:**
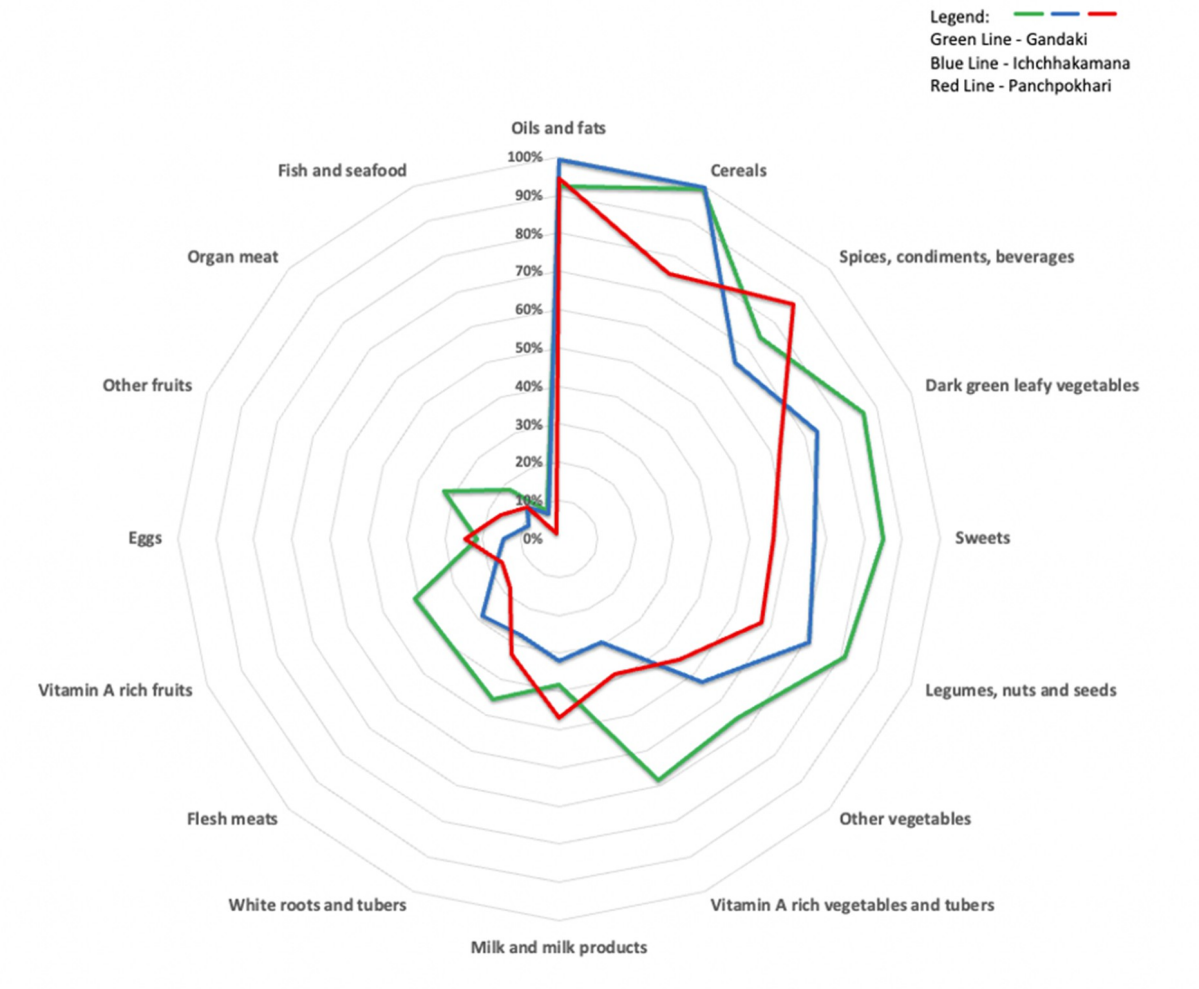
Percentages of households that consumed different food items.

The sample households consumed mostly oils and fats (95% of the total households), cereals (90%), spices and condiments (78%), dark green leafy vegetables (74%), sweets (69%), and legumes and nuts (69%). The less consumed food items were fish (5% of all households), organ meat (14%), other fruit (21%), eggs (21%), vitamin A-rich fruit (26%), flesh meats (29%), and white roots and tubers (36%). Municipalities vary widely in food items consumed (Fig 2). For example, dark green leafy vegetables were consumed mainly in Gandaki (86% of households), compared to the households in Ichchhakamana (73%) and in Panchpokhari (63%). Likewise, vitamin A-rich fruit consumption was reported more in Gandaki (41% of households) than in Ichchhakamana (18%) and Panchpokhari (16%). These results are in line with a long-term study that found increased consumption of oils and fats by manyfold in about 4 decades, while the share of staples like cereals and tubers was slowly declining [34].

### 3.5 Dietary diversity scores

**Fig 3:**
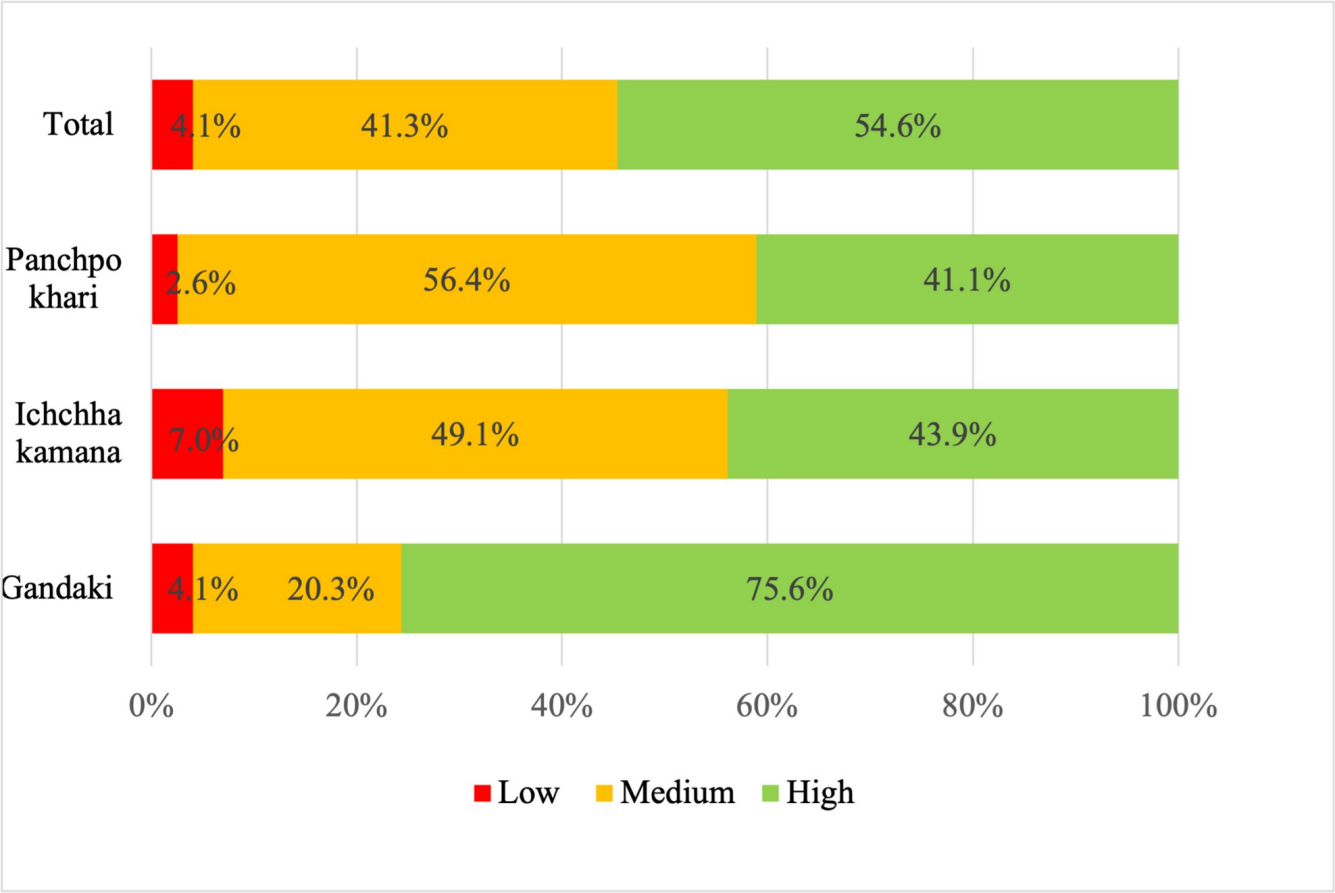
Percentage of Households under low, medium, and high HDDS groups. HDDS = household dietary diversity score.

Both the HDDS and the WDDS were lowest in Panchpokhari municipality and highest in Gandaki (Table 10). Frequency analysis shows that one household in Gandaki and Panchpokhari had no food groups rich in micronutrients in their household diet (WDDS = 0). The HDDS categorization following Mekuria et al. (2017) [33] shows that almost 76% of the households in Gandaki had a “high” HDDS compared to 44% in Ichchhakamana and 41% in Panchpokhari (Fig 3).

**Table 10:** Estimated dietary diversity score.

Municipality	Household Dietary Diversity Score (HDDS)				Women’s Dietary Diversity Score (WDDS)			
	Min	Max	Mean	SD	Min	Max	Mean	SD
Gandaki	2	12	7.44	1.86	0	9	5.34	1.47
Ichchhakamana	3	11	6.27	1.72	2	8	4.26	1.38
Panchpokhari	1	11	6.22	1.76	0	8	4.00	1.56
Total	1	12	6.69	1.88	0	9	4.56	1.61

Min = minimum, Max = maximum, SD = standard deviation.

### 3.6 Factors influencing the dietary diversity scores

We provide the summary statistics of independent variables used in the OLS regressions in S2 Table, showing that our sample population was middle aged, one-third were men, mainly the household head, and having relatively larger household size. The dependent variables are the HDDS and the WDDS, while independent variables are grouped into individual and household characteristics of respondents (Table 11).

**Table 11:** Regression results—factors influencing dietary diversity scores.

	Household dietary diversity score (HDDS)			Women’s dietary diversity score (WDDS)		
Variables	Unstandardized Coefficients		Standardized Coefficients	Unstandardized Coefficients		Standardized Coefficients
	Coeff	Std. Error		Coeff	Std. Error	
Constant	1.442**	0.542		2.002**	0.470	
Age of respondent (years)	0.019**	0.004	0.159	0.006*	0.003	0.063
Gender of respondent (male = 1)^a,b^	0.120	0.132	0.030	0.105	0.114	0.030
Respondent as the household head^a,c^	-0.618**	0.125	-0.164	-0.463**	0.108	-0.144
Household size	-0.037*	0.019	-0.058	0.009	0.016	0.016
Household asset index	0.299**	0.035	0.241	0.201**	0.030	0.190
Location 1 (Gandaki)^a,d^	0.602**	0.151	0.155	0.840**	0.131	0.252
Location 2 (Panchpokhari)^a,d^	-0.021	0.158	-0.005	-0.273*	0.137	-0.083
Lowland *(Khet)* (low land with irrigation)	0.313*	0.142	0.065	0.130	0.123	0.032
Upland *(Bari)* (sloped upland without irrigation)	-0.237**	0.090	-0.070	-0.097	0.078	-0.034
Upland *(Bari)* land abandonment^a^	-0.368**	0.139	-0.072	-0.345**	0.120	-0.079
Food sufficiency^a,f^	0.344*	0.134	0.075	0.648**	0.116	0.164
Frequency of food purchase from market	0.601**	0.138	0.116	0.206	0.120	0.047
Livestock unit 1(dairy)	0.016	0.071	0.006	-0.033	0.061	-0.015
Livestock unit 2 (eggs and meat)	0.074**	0.038	0.054	0.094**	0.033	0.080
Log remittance income	0.195**	0.076	0.071	0.028	0.066	0.012
Index for women’s involvement in household chores	0.222**	0.092	0.076	0.096	0.080	0.038
Index for women’s involvement in community	0.366**	0.047	0.216	0.182**	0.040	0.126
Model summary	N = 1053, R^2^ = 0.297, Adjusted R^2^ = 0.285, Standard Error of Estimate = 1.592, F test = 25.72, p<0.001			N = 1053, R^2^ = 0.279, Adjusted R^2^ = 0.267, Standard Error of Estimate = 1.380, F test = 23.52, p<0.001		

Notes: * and ** indicates significance at the 1% and 5% level of significance, respectively. ^a^Dummy variable, ^b^basecategory = female ^c^basecategory = if respondent is not head ^d^basecategory = Ichchhakamana; ^e^basecategory = household own food production not sufficient for 12 months.

#### Effects of individual characteristics

Elderly respondents were likely to have higher HDDS and WDDS, which is against our initial hypothesis proposed in Table 4. This is possibly because elderly respondents accumulate wealth over time and can diversify their diets. However, as expected, household heads reported minimum dietary diversity for women and for households (both WDDS and HDDS). One possible reason could be the involvement of young women in cooking [35]. Whereas household heads, generally older males, are not involved in food preparation, and thus missed some food items to include in the (previous 24 hours) meals.

#### Effects of farm size, household assets, and location

The HDDS was higher in small and nuclear households and lower among the households with larger family size, but this was not true for the WDDS. Previous studies also reported similar results [36, 37]. The dietary diversity increased with an increase in the household’s economic status, as indicated by the positive and significant coefficient of asset ownership. In the study area context, affluent households own more natural capital such as land, especially high-value and productive land, i.e., irrigated lowland *(khet)*. At the same time, these well-off households have better opportunities to increase consumption expenditures on food items, especially the ones bought from the market. As asset ownership (land, farm machinery) contributes to higher production and production diversity therein [38] these economically well-off households have the potential to increase dietary diversity. Households in Gandaki are located near the national highway and urban markets; hence, they can access a variety of food to diversify their diet (Table 1). In contrast, households in Panchpokhari had significantly low WDDS because of the limited opportunity to grow and buy diverse food. Limited access to technology, seeds, markets, and road connectivity are some factors that hinder households in relatively remote areas (Panchpokhari) from diversifying their diet. Hirvonen and Hoddinott [39] emphasized the role of local geography in the access to food based on the crops grown in the areas, determining the diversity of food and diet.

#### Effects of land and food dynamics

The size of irrigated lowland (*khet)* plots was positively associated with the HDDS and the WDDS, while unirrigated upland (*bari)* was negatively associated. However, their effect was statistically significant only on the HDDS and not on the WDDS. In the hills and mountains of Nepal, lowland plots *(khet)* have a relatively higher value due to higher productivity and the potential to grow diverse crops and sell surplus production. As stated by Dahal et al. [40], “[lowland] *khet* received a net deposition of nutrients washed down from the upland sloping areas,” implying that upland *(bari)* plots are subject to nutrient loss and erosion during rainfall. Lowlands *(khet)* are relatively more productive and easier to work, leading farmers to intensify the cultivation of cereals, pulses, vegetables, and tubers. On the other hand, uplands *(bari)* are rainfed and not connected to irrigation facilities, and hence less productive. As non-staples and farm income are important in improving both individual and household dietary diversity [41], owning a large lowland area *(khet)* would increase the HDDS. Small upland *(bari)* plots used as kitchen gardens and orchards can diversify crops, which also helps to diversify diets. However, large upland *(bari)* plots negatively affect crop diversity and the HDDS because such land is used to produce cereals such as maize, millet, buckwheat, and some tubers, hence less opportunity for crop diversity.

Results showed lower HDDS and WDDS when migrant households hold larger upland *(bari)* plots. Households in the hills and mountains seldom allocate entire upland *(bari)* plots for cultivating diverse crops because of labor shortages and other limitations, including low soil fertility, erosion, steep slopes, remoteness, and lack of irrigation. Furthermore, crop cultivation on upland *(bari)* plots is constrained by the acute shortage of male labor. Participants in the women focus group discussions confirmed that left-behind women usually need to invest remittances in hiring oxen plows for land preparation, costing them about NPR2,000 per day (about US$18 [US$1 = NPR110 at the time of the survey]), which increases the cost of production. They further reported that oxen power is mandatory for tillage operation as upland *(bari)* plots are too steep for other farm implements, like tractors. Due to labor constraints, migrant households own less livestock; hence, the migrant households rely on the hired oxen and men’s power. When there is no money to hire male labor and oxen for land tilling, households sometimes abandon land parcels, especially the distantly located upland *(bari)* plots. In support of this fact, a recent study states that around 40% of agricultural land in their sample had been left fallow, and about 60% of farmers abandoned at least a portion of land used for cultivation [42], which may happen more in migrant households where women have greater decision-making power [43, 44]. Land abandonment in the hills and mountains of Nepal is growing significantly and affecting household food security and environmental degradation [45]. This trend might have changed recently; the key informants mentioned that because of the COVID-19 pandemic, many migrant returnees cleared and worked the abandoned land and started re-establishing farms, thereby contributing to household food security.

Households that are not food secure from their own production are less likely to have adequate dietary diversity considering the poor economy and low purchasing power of Nepali farming households [46]. Our regression results show that food self-sufficient households have higher HDDS and WDDS. Those households are also more likely to have higher farm production diversity, as a positive association between farm production diversity and household dietary diversity is evident [13, 47].

Households with a higher frequency of food purchase had higher HDDS and WDDS, indicating that households closer to the market can purchase nutritious and diverse food. The improved market interaction aided by remittances can reduce food insecurity. For instance, studies conducted elsewhere observed an improved HDDS through market access and interaction [41, 48] because of the diverse food basket [20], however Kim et al. [6] reported that market interaction did not bring any improvement in dietary diversity and quality for the left-behind households in far western Nepal.

The literature shows women’s increased access to diverse diets due to improved market infrastructure and the opportunity to purchase food; however, the studies have cautioned that the purchased food might not be nutritious enough [49]. This highlights that food purchases can improve the HDDS but may not ensure the consumption of nutritious food groups as included in the WDDS. It is also possible that with increasing market interaction, consumption of processed foods with higher total energy, fat, and sugar content increases as well [34]. Hence, we argue that although access to the market could increase household dietary diversity, it may not add adequate nutrition to the diet. It is less likely that purchased food items are healthy and nutritious. To establish a definitive relationship, we suggest further research on the nutritional aspect of the food purchased and consumed by rural migrant households.

#### Effects of large and small livestock

There is a complex relationship between agriculture, farm animals, and household food security. Farm animals provide farmyard manure and support households in generating income and improving dietary diversity. Livestock ownership, an important asset for farm households, is linked with positive annual net income through marketable surplus and frequent consumption of milk and dairy products, and meat compared to households with less livestock [50]. At the same time, income from the sale of livestock products also increases the household’s consumption expenditure [51]. Thus, it can be said that livestock ownership enhances the HDDS and the WDDS. Our regression results show that the relationship between livestock unit 1 (index for milk and dairy products) and dietary diversity is not straightforward. Although statistically nonsignificant, we observed its positive influence on the HDDS, but negative on the WDDS. One possibility is that the allocation of milk and dairy products is different for the household members based on their choice, need, or family hierarchy [52, 53]. However, we observed a significant positive association between livestock unit 2 (meat and eggs) and the HDDS and the WDDS. This illustrates that having large farm animals such as cows and buffaloes may not always enhance dietary diversity, while raising small farm animals such as poultry and goats improves both the HDDS and the WDDS.

#### Effects of migration and women’s agency

It is evident that remittances from out-migration significantly impact the HDDS, but not the WDDS. Higher the remittances, higher is the HDDS because it provides an opportunity to purchase diverse food for home consumption, but it may not correspondingly increase the WDDS because food purchased from markets may not have adequate and diverse nutrition. Similar results were observed in a study among migrant households in other parts of Nepal, where remittances contributed to enhanced food availability but not nutrient-rich dietary intake [6]. Our findings further support the notion that the use of remittances for the purchase of quality food for home consumption is minimal.

A group of scholars [6, 54, 55] argues that out-migration increases the work burden of women, especially in household chores and farm activities; whereas others such as Maharjan [56] argue that remittances, if received in larger amounts, can offset such an increased work burden of women. In many contexts, women are found to bear the household and farm work burdens, but still have an inferior spot for intrahousehold food allocation and decision making. Women members of a household are served last or after men and face cultural restrictions to food in many households in Uganda [52, 57]. We found that migrant households with women’s increased involvement in household chores had a significantly higher HDDS, but not a higher WDDS. In contrast, migrant households with women’s increased involvement in community work had significantly higher HDDS and WDDS. This concurs with other studies that observed women’s participation in community work helps them to expand their agency and supports them in becoming empowered [20, 54, 58–60]. Women’s agency positively influences the household dietary diversity [25] but can be limited when daughters-in-law live in an extended family [58]. Yet, in patriarchal societies, major household decisions are always made by men, and in some cases, even a simple decision as to what crop to sell and what to consume needs a man’s approval [61, 62]. Nevertheless, despite these complex intrahousehold dynamics that need further research, there is a common consensus that women with expanded agency are more likely to ensure higher dietary diversity in the households [23, 24] although not in every context [25].

Therefore, factors like land ownership and farm production, asset ownership, higher livestock unit, remittances, and migration are important considerations for a better HDDS. However, it is equally critical to understand the intrahousehold dynamics and food allocation to ensure nutrition security for all household members. Food allocation within a household may differ based on age, relationship, marital status, gender, occupation, and other sociocultural factors [49, 63, 64]. Although women may have some level of bargaining power to access food in general [52, 62], they are usually the ones who eat last during a meal, often with the leftovers. This implies that, in the cases of food shortages, they are left with no option [49, 57], resulting in low dietary diversity and low micronutrient uptake [65]. On this point, we argue that the studies solely based on the HDDS do not effectively ensure nutrition security, especially in unraveling the intrahousehold gender food allocation. Instead, as Obisesan and Awolala [66] suggested, farm diversification and women’s empowerment to improve their agency could be a better option. At the same time, even though out-migration increases women’s agency while men are away, it is doubtful that the change is permanent. Hence, we recommend further research on the interrelationship between remittance flows and intrahousehold gender dynamics, especially on the long-term change in women’s agency and intrahousehold food distribution.

## 4. Conclusion and policy implications

This study explores a deeper understanding of the migration–diet–gender nexus at both the household and individual levels. The nexus is complex not only because of the interplay among several social, economic, and geographical factors, but also because of the intrahousehold gender roles and relations. Dietary intake depends on the relationship of women with other household members, gender and social norms, women’s hierarchal status in the household, and cultural restrictions and social taboos around food consumption, which are often biased against women [53]. In the male labor out-migration context, women’s access to remittances and the use of remittances for daily consumption affect household dynamics and food distribution [67]. Dietary intake may differ at an individual level, but most studies have only focused on the household level.

We establish the relationship of upland *(bari)* and irrigated lowland *(khet)* ownership with dietary diversity, and call for further investigation to empirically examine the effects of farmland abandonment on household and individual dietary diversity. Likewise, holdings of large farm animals, such as cows and buffaloes, contributed very little to a household’s dietary diversity. However, raising small farm animals like poultry and goats contributed more to the household’s dietary diversity. Direct consumption of animal products and increased income from the sale of small farm animals could justify the higher household dietary diversity. Women’s empowerment contributed to enhanced dietary diversity; however, their families lack a nutritious diet when women are confined to household chores.

Moreover, economic status is positively related to dietary diversity, directly supporting food security outcomes [37]. Women’s empowerment opportunities, livestock and productive irrigated lowland *(khet)* ownership, as well as the purchasing power to diversify the diet, are closely related with the socioeconomic status and intrahousehold dynamics [37]. The food consumption share of remittance income might also differ according to the economic status, contributing to differences in the nutritional value of food purchased. This implies that integrated farming system models with built-in women’s empowerment packages could lead to better dietary diversity among migrant households in Nepal. We further suggest that the formulation of policies at local and national levels should build on women’s agency together with social, economic, and market interventions for adequate and equitable food and nutrition security.

Finally, we would like to admit a few caveats of the study. We adopted a 24-hour recall period and used the HDDS and the WDDS for dietary diversity measurement at the household level. There are other methods for such measurement as well, for example, food consumption score and minimum dietary diversity of women. Our study does not provide the quantity of food consumed at the household or individual level, and it only offers qualitative measures of household dietary diversity. The literature suggests a great seasonal variation in the availability of household diet. For example, festivals and harvesting seasons can significantly affect the frequency, amount, and variety of food groups consumed.

## Supporting information

S1 Table Household diet (24 h) home consumption. (DOCX)

S2 Table Summary statistics of the independent variables used in the regression analysis. (DOCX)
