# Supplementary material for: Factors influencing household and women’s dietary diversity in migrant households in central Nepal
Source: PLoS One. 2024 Apr 5;19(4):e0298022. doi: 10.1371/journal.pone.0298022 (PMC10997064; doi:10.1371/journal.pone.0298022)
Supplement: S1 Table — (DOCX) [file pone.0298022.s001.docx]

# **Factors Influencing Household and Women’s Dietary Diversity in Migrant Households in Central Nepal**

**S1 Table. Household diet (24 h) home consumption**

| Food group | Example | Photos |
| --- | --- | --- |
| Cereals | Cereals like rice, maize, millet, wheat, buckwheat, and the food items prepared from these or other cereals. Example: bhat (steamed rice), kheer (rice), chyakhla (maize), dhindo (millet dough), chapati/roti (wheat/rice), popcorn (maize) etc. | 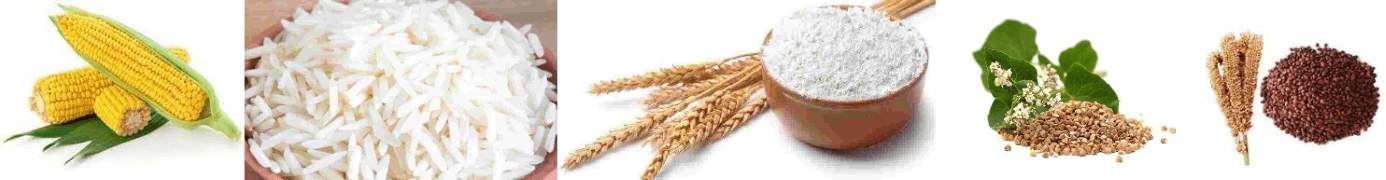  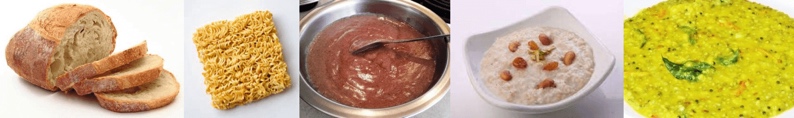 |
| White tubers and roots | White potato, white sweet potato, white yam, squash root etc., and the items prepared from them. | 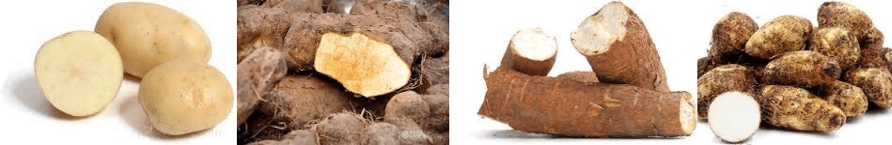 |
| Vitamin A rich vegetables and tubers | Vitamin A rich vegetables like pumpkin, carrots, squash, orange-colored tubers, red bell pepper, broccoli etc. | 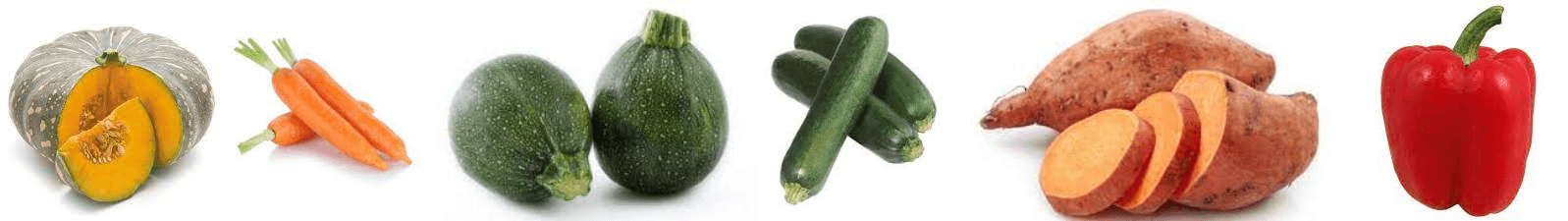 |
| Dark green leafy vegetables | Both homes grown and foraged dark green leafy vegetables. Example: mustard, rapeseed greens, buckwheat greens, green onion, lettuce, coriander, bok choy leaves etc. | 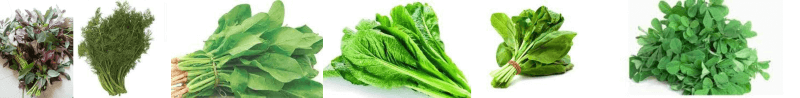 |
| Other vegetables | Other vegetables like tomato, onion, brinjal, cauliflower, cabbage, locally available vegetables. | 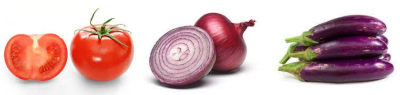 |
| Vitamin A rich fruits | Ripe mango, papaya, peach, watermelon, kiwifruit etc., and 100% drinks/juice prepared from these fruits. | 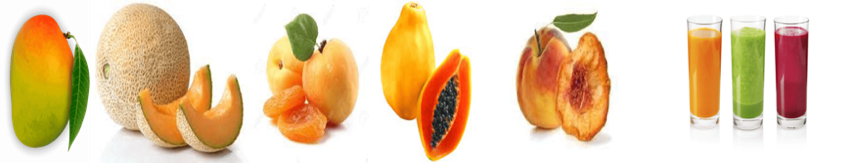 |
| Other fruits | Other fruits such as orange, guava, pears, wild berries etc., and 100% drinks/juice prepared from these fruits. | 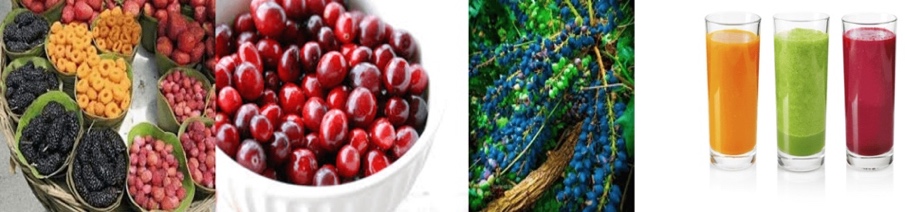 |
| Orange meat | Heart, liver, kidney, or other organ or blood related meat items. | 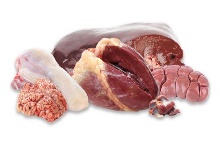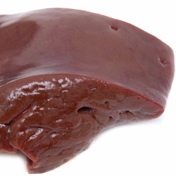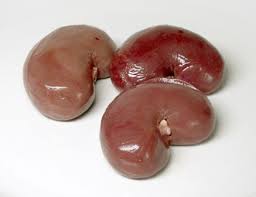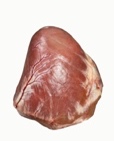 |
| Flesh meat | Goat, pig, buffalo, chicken, duck, pigeon, quail meat, or meat of any other animal/birds/insects. | 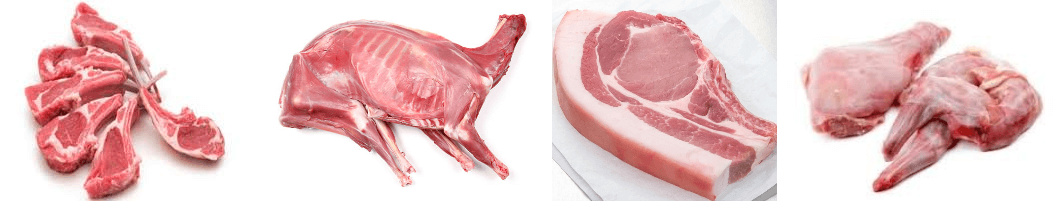  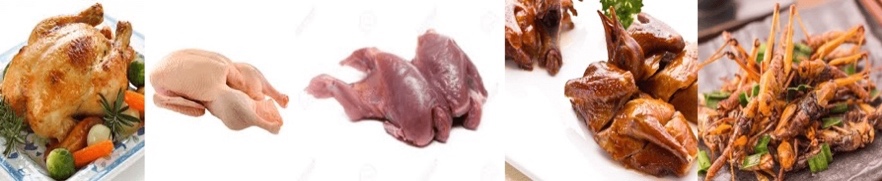 |
| Eggs | Eggs from chicken, duck, pigeon, quail etc. | 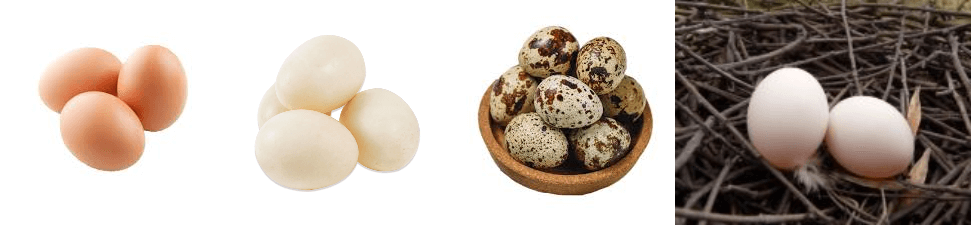 |
| Fish and seafood | Fresh or dried fish, prawn, octopus, snail etc. from freshwater and/or sea. | 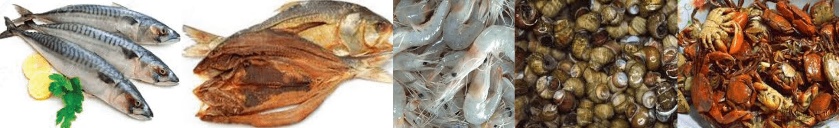 |
| Legumes/nuts/seeds | Dried beans, peas, chickpeas, black gram, horse gram, ground nuts pumpkin seeds, perilla seeds (silaam) etc., paste, or pickle prepared from these seeds. | 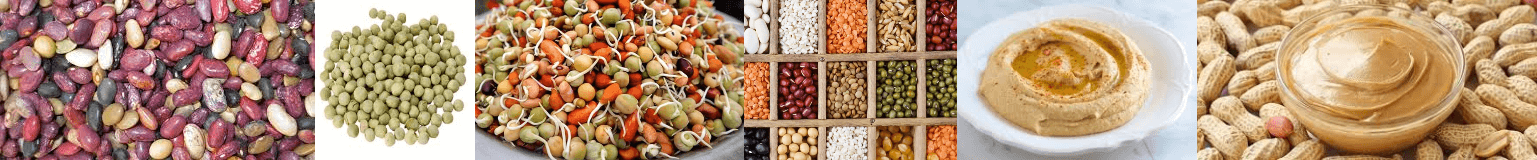 |
| Milk and milk products | Milk, cheese, yogurt, khuwa, and other food items derived from milk | 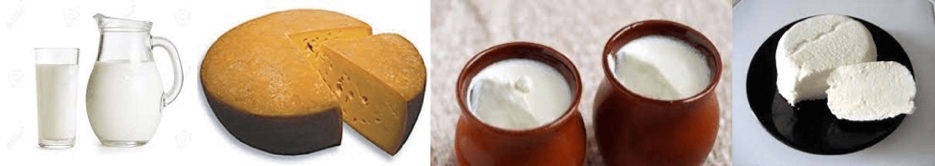 |
| Oil/fats | Oil, butter, ghee, other animal-based fat, foods prepared using any kind of oil/fat, oil fried items like *Selroti* or *Furaula*. | 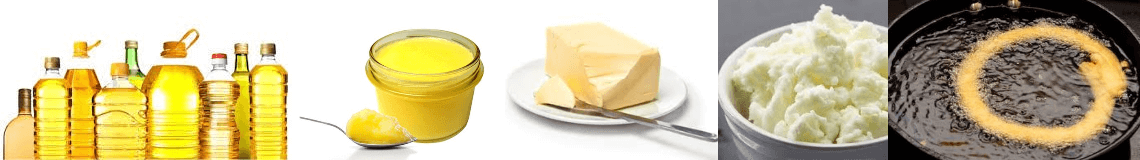 |
| Sweets | Sugar, raw sugar (sakhkhar), honey, chocolates, candy, coke, cake Indian/Nepali sweet items like laddu, peda, rasbari, anarasa, arsaa etc. | 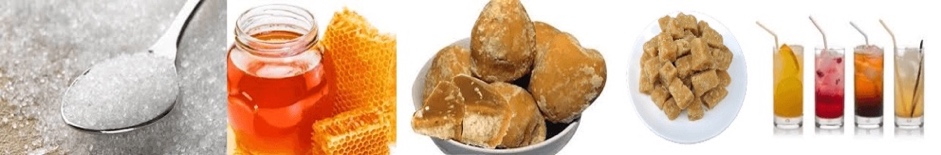  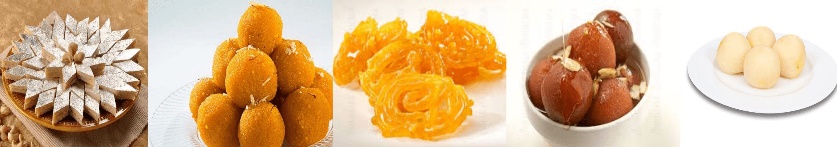 |
| Spices, condiments and beverages | Black pepper, cardamom, cumin and coriander powder, salt, soya sauce, hot sauce, tea, soft drinks, and alcohol-based drinks etc. | 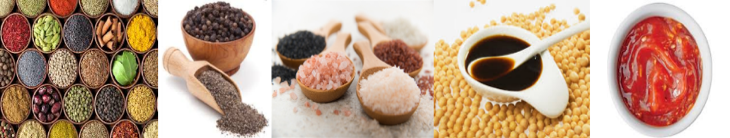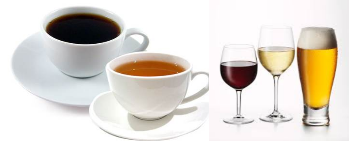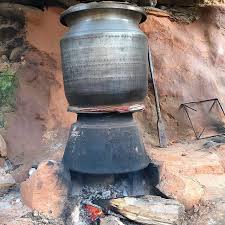 |

Source: Adapted from [1]; modified for the locally available food sources.


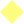


**References**

1. FAO. Guidelines for measuring household and individual dietary diversity. Rome, Italy: Food and Agriculture Organization; 2010.
