# Supplementary material for: Factors influencing household and women’s dietary diversity in migrant households in central Nepal
Source: PLoS One. 2024 Apr 5;19(4):e0298022. doi: 10.1371/journal.pone.0298022 (PMC10997064; doi:10.1371/journal.pone.0298022)
Supplement: S2 Table — (DOCX) [file pone.0298022.s002.docx]

# **Factors Influencing Household and Women’s Dietary Diversity in Migrant Households in Central Nepal**

**S2 Table. Summary Statistics of the Independent Variables used in the Regression Analysis**

| **SN** | **Variable** | **Mean** | **SD** |
| --- | --- | --- | --- |
| 1 | Age of respondent (in years) (C) | 44.59 | 15.88 |
| 2 | Gender of respondent [male=1, female=0] (D) | 0.33 | 0.47 |
| 3 | Respondent as the household head (D) | 0.52 | 0.50 |
| 4 | Household size (C) | 6.51 | 2.92 |
| 5 | Household asset index (C) | 3.22 | 1.52 |
| 6 | Location 1 Gandaki (Base category – Ichchhakamana) (D) | 0.37 | 0.48 |
| 7 | Location 2 Panchpokhari (Base category – Ichchhakamana) (D) | 0.41 | 0.49 |
| 8 | Lowland *(Khet)* (lowland with irrigation) in hectares (C) | 0.21 | 0.39 |
| 9 | Upland *(Bari)* (sloped upland without irrigation) in hectares (C) | 0.31 | 0.56 |
| 10 | Upland *(Bari)* abandonment (D) | 0.16 | 0.37 |
| 11 | Food sufficiency (D) | 0.21 | 0.41 |
| 12 | Frequency of food purchases from the market (C) | 2.05 | 0.37 |
| 13 | Livestock unit 1(dairy animals) (C) | 0.58 | 0.72 |
| 14 | Livestock unit 2 (livestock for eggs and meat) (C) | 0.69 | 1.37 |
| 15 | Log remittance income (C) | 2.14 | 0.69 |
| 16 | Index for women’s involvement in household chores (C) | 3.68 | 0.64 |
| 17 | Index for women’s involvement in the community (C) | 2.67 | 1.11 |

SD= Standard deviation, (C) = Continuous variable, (D) = Dummy variable.
